# Rapid, multimodal, critical care knowledge-sharing platform for COVID-19 pandemic

**DOI:** 10.17305/bjbms.2020.4934

**Published:** 2021-02

**Authors:** Amra Sakusic, Dragana Markotic, Yue Dong, Emir Festic, Vladimir Krajinovic, Zoran Todorovic, Alan Sustic, Natasa Milivojevic, Milka Jandric, Srdjan Gavrilovic, Alexander S Niven, Pedja Kovacevic, Ognjen Gajic

**Affiliations:** 1 Department of Medicine, Division of Pulmonary and Sleep Medicine, Mayo Clinic, Jacksonville, Florida, USA; 2 Multidisciplinary Epidemiology and Translational Research in Intensive Care, Emergency and Perioperative Medicine (METRIC), Mayo Clinic, Rochester, Minnesota, USA; 3 University Clinical Hospital Mostar, Bosnia and Herzegovina; 4 Department of Anesthesiology and Perioperative Medicine, Mayo Clinic, Minnesota, USA; 5 Hospital for Infectious Diseases, Zagreb, Croatia; 6 Department of Pharmacology, Clinical Pharmacology, and Toxicology, School of Medicine, University of Belgrade, Belgrade, Serbia; 7 University Medical Center “Bezanijska kosa”, Belgrade, Serbia; 8 Department of Anesthesiology, Reanimatology, Emergency and Intensive Care Medicine, University of Rijeka, Croatia; 9 Department of Neurology, University Medical Centre Ljubljana, Slovenia; 10 Medical Intensive Care Unit, University Clinical Centre of the Republic of Srpska, Banja Luka, Bosnia and Herzegovina; 11 Institute for Pulmonary Diseases of Vojvodina, Sremska Kamenica, Serbia; 12 Faculty of Medicine, University of Novi Sad, Novi Sad, Serbia; 13 Department of Medicine, Division of Pulmonary and Critical Care Medicine, Mayo Clinic, Rochester, Minnesota, USA

**Keywords:** COVID 19, intensive care unit, critical illness, education, knowledge sharing, global health

## Abstract

In many areas of the world, critical care providers caring for COVID-19 patients lacked specific knowledge and were exposed to the abundance of new and unfiltered information. With support from the World Health Organization, we created a multimodal tele-education intervention to rapidly share critical care knowledge related to COVID-19 targeting providers in a region of Southeastern Europe. We delivered 60-minute weekly interactive tele-education sessions over YouTube™ between March and May 2020, supplemented by a dedicated webpage. The intervention was reinforced using a secure social media platform (Viber™) providing continuous rapid knowledge exchange among faculty and learners. A high level of engagement was observed with over 2,000 clinicians participating and actively interacting over a six-week period. Surveyed participants were highly satisfied with the intervention. Tele-education interventions using social media platforms are feasible, low-cost, and effective methods to share knowledge during the COVID-19 pandemic.

## INTRODUCTION

On January 30, 2020, the World Health Organization (WHO) officially declared the COVID-19 pandemic. More than 38.5 million cases have been confirmed and more than million patients have died as of October 15, 2020 [1]. Severe acute respiratory syndrome coronavirus 2 (SARS-CoV-2), the strain of coronavirus that causes COVID-19, is definitely the greatest global healthcare crisis in the last 100 years. In response to this new and largely unknown threat to modern society, there has been an abundance of information, much with little or no vetting through traditional peer review processes.

Healthcare workers, already stressed by large numbers of patients, insufficient supplies, and infection control concerns, have struggled to synthesize useful information from these information sources into practical recommendations that can be readily implemented at the bedside. These challenges have been even more formidable in low- and middle-income countries, where the providers had limited critical care training and experience [2]. WHO has identified education as a major ­priority in these settings, but distance and resources remain significant barriers to rapid dissemination [3]. Structured tele-education critical care programs have previously been shown to be effective in overcoming these barriers [4]. A video-enabled remote simulation training based on a structured platform (CERTAIN: Checklist for Early Recognition and Treatment of Acute Illness) [5] has proved to be an efficient educational method to share clinical knowledge among critical care practitioners in diverse international settings [3]. Following the request from a WHO office from Sarajevo, Bosnia and Herzegovina, this program was modified to incorporate best available practice recommendations for the critical care of COVID-19 patients and delivered using internet-based web resources and a social media platform to a target audience of healthcare providers in Bosnia and Herzegovina and other countries of former Yugoslavia. The aim of this study was to evaluate the feasibility and reach of this intervention, as well as participants’ engagement and satisfaction.

## MATERIALS AND METHODS

Within one week of the initial WHO request, we organized a dedicated webpage, secure chat, and conducted the first in the series of tele-education interventions weekly between March 24^th^ and May 2^nd^, 2020. Each education session included expert intensivists from Mayo Clinic in Rochester, Minnesota (OG), and Jacksonville, Florida (EF), who are both proficient in the local languages (Bosnian-Croatian-Serbian) and served as educators, in collaboration with experts from across Southeastern Europe in different subspecialties (including Anesthesiology, Infectious Diseases, Pharmacology, Cardiology, Pulmonary, and Neurocritical Care) at a time that was convenient for all parties. Viber™ (Rakuten, Inc.) secure messaging service was used to connect the healthcare providers who participated in this intervention as learners, enabling them to ask questions on a 24/7-hour basis given time-zone differences. Zoom™ (Zoom Video Communication, USA) was used for live online meetings, during which faculty members discussed important and emerging clinical themes related to the care of critically ill COVID-19 patients and answered questions related to their everyday management. Clinicians were able to follow the meetings on YouTube™ live and ask questions via Viber™. These interactive sessions were recorded and stored on the project web page to provide further opportunities asynchronous viewing on demand. During each tele-education session, we used a standardized, systematic, organ system-based patient presentation algorithm. The participants were also provided pragmatic, curated content on current COVID management recommendations, ventilator management, and appropriate use of personal protective equipment through a public web page (https://www.icertain.org/covid). Asynchronous learning and spaced reinforcement were provided through ongoing Viber™ discussions on the overall management and critical care of COVID-19 patients. No names or other personal identifiers were used during Zoom™ presentations or Viber™ discussions in order to protect patient privacy and confidentiality.

After the fourth and fifth tele-education interventions, we conducted an online anonymous survey (Table 1) using an electronic Google™ form posted on Viber chat group to evaluate participants’ profession, specialty, their satisfaction, and viewpoints about the content in the program, as well as the platforms applied. We also collected anonymous visitor’s data from YouTube™, Viber™, and total visits to the site using web analytics tools to measure participants’ engagement, including time spent watching educational materials and the number of messages, views, likes, and shares of the educational content.

**TABLE 1:**
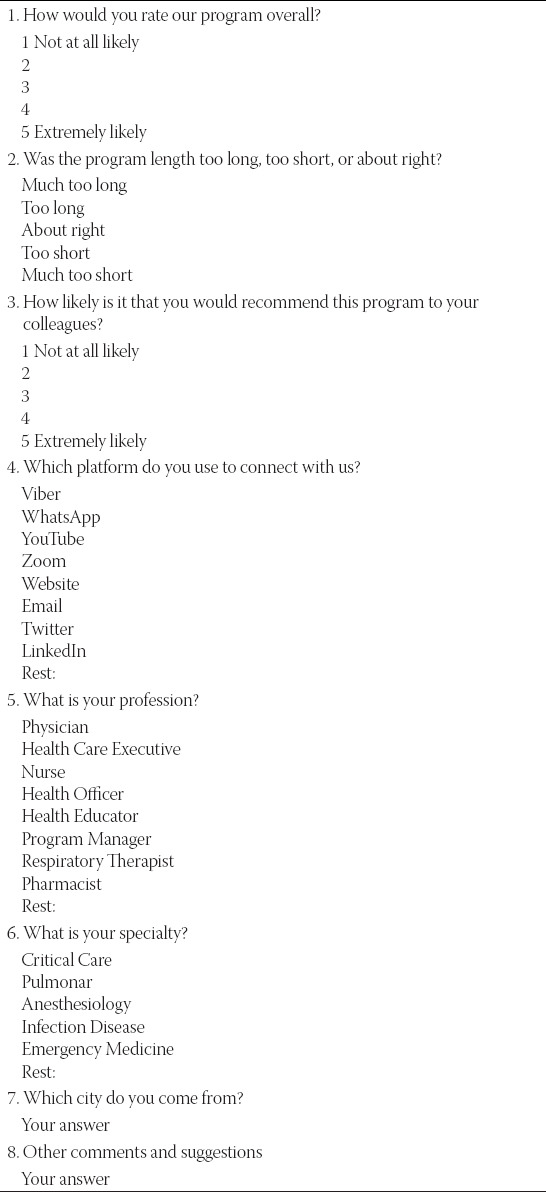
2020 CERTAIN WHO survey

## RESULTS

**Figure 1:**
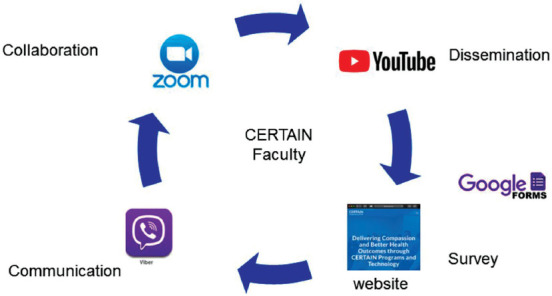
Schematic representation of the multimodal knowledge-sharing platform.

A summary of the multimodal educational intervention is depicted in Figure 1. Six COVID-19 tele-education sessions were conducted on Zoom™ and broadcasted on YouTube™ between March 24^th^ and April 27^th^, 2020. By May 12^th^, 2020, 2,096 participants had joined the Viber™ WHO CERTAIN ICU Support Group, with 31 faculty members. Between April 6^th^ and May 12^th^, 2020, 1,990 educational messages were exchanged on Viber, followed by 12,443 likes and 297,226 views. Participants were from 25 countries, with the highest engagement of clinicians from Serbia, Bosnia and Herzegovina, United States, Croatia, North Macedonia, and Montenegro.

Learners spent a cumulative 2,143.9 hours watching tele-education sessions on YouTube™, mostly via mobile phones. The average visit duration was 15 minutes. From March 15^th^ to May 9^th^, 2020, there were total 4,752 visits from 4,120 unique visitors to YouTube™ tele-education sessions with 8,658 page views.

**FIGURE 2:**
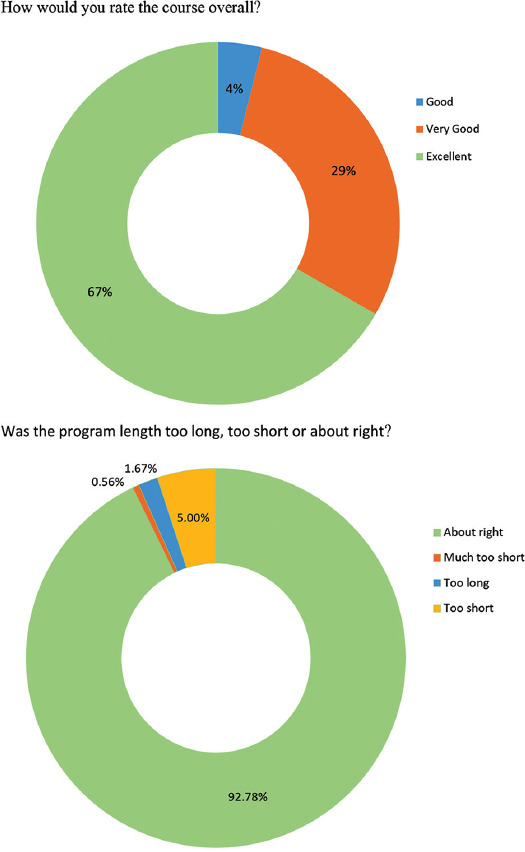
Survey results.

Participants were invited on an anonymous survey on 2 occasions after YouTube sessions on April 14^th^ and April 27^th^, 2020, that were followed by 425 and 192 unique participants, respectively. One hundred and eighty of them responded to survey (29.2% response rate), 162 following the 1^st^ invitation and additional 18 following the 2^nd^ invitation. Survey results indicated a high degree of learner satisfaction, majority of the learners rated the course excellent (66.7%) or very good (29.4%), and 92.8% of learners rated the program length was about right (Figure 2). More than 90% of survey respondents were physicians, predominantly from the specialties of anesthesiology and critical care.

## DISCUSSION

The COVID-19 pandemic has overwhelmed critical care facilities and healthcare providers in many different areas of the globe. When treating a novel disease of such magnitude, physicians were faced with many unanswered questions and challenged to identify best practice recommendations amid a rapidly growing body of largely non-peer-reviewed information of variable quality. Although there have been many papers related to COVID-19 published online, it has been challenging for frontline providers to synthesize this information into streamlined, practical guidelines for treatment. To avoid the well-recognized, common delays in the implementation of best practice recommendations at the bedside [6,7], an expert-guided approach to facilitate knowledge translation into practice is crucial to avoid information overload and to enable streamlined evidence-based care delivery. In our project, majority of faculty members was familiar to CERTAIN method which has previously been shown to improve the performance of clinical providers faced with simulated emergencies [3,5]. Challenged with an unknown virus, clinical presentation, diagnosis and therapeutic options, as well as persistent new data with a questionable level of evidence, original CERTAIN method was modified to meet the specific demands of the pandemics. Topics were broadened to cover all important issues about SARS-CoV-2 virus, including pathogen ­characteristics, patient and healthcare providers’ protection, and specific treatment recommendations. In order to accomplish that, faculty from different specialties participated in tele-education sessions and as moderators on social media platform. Using the essentials of CERTAIN methodology in a way of the standardized, systematic and structured approach to virtual COVID-19 patient proved to be a suitable framework to cover all important and newly emerging issues relevant to the problem. Low- and middle-income countries routinely confront additional challenges because of their limited critical care capabilities, less well-developed critical care teams, and access and language barriers to the high-impact literature. The WHO has advocated for increased educational efforts using simulation, telemedicine, and Internet-based courses for healthcare workers in low- and middle-income countries [3]. Our study demonstrated that tele-education interventions using readily available and low-cost video conference and social media platforms can offer an inexpensive and rapid method to share knowledge in the midst of a global pandemic, with 96% of survey participants rating the program as excellent or very good. Participants’ engagement, judged from the extent of interaction was evaluated by quantity of educational message interactions, followed by likes and views as well as by number of hours spent watching tele-education sessions and total visits to the CERTAIN website. Additionally, very high number of unique YouTube visitors demonstrated that the material was frequently shared outside the Viber chat group, which also reflects the high level of engagement and satisfaction.

Previously published studies [8] show that social media is being increasingly utilized as a resource in medical education. Healthcare practitioners alike consider it to be an effective educational tool [8]. The previous implementation of tele-medicine intervention based on the CERTAIN program showed it to be feasible, well-received and associated with significant improvement in clinical outcomes and costs [4]. CERTAIN was also used as a part of tele-medicine intervention implemented in New York City ICUs overwhelmed by pandemics [9]. Rapid implementation, high reach, and engagement suggest that this method is highly convenient in global emergencies such as this coronavirus outbreak. The effectiveness of the intervention, however, cannot be evaluated without formal knowledge assessment and baseline and follow-up data on processes of care and outcomes. Importantly, tele-education interventions may offer a particularly favorable alternative to in-person educational efforts in resource-limited settings [5]. These interventions can effectively fill knowledge gaps and provide expert advice where local expertise is limited.

Our study has several limitations. Due to the rapid deployment of this intervention and out of respect for the limited resources and time available to many of our program participants, we only measured the clinician engagement and satisfaction. Further studies, including international VIRUS registry (https://sccmcovid19.org/), are needed to more rigorously measure the impact of this and similar educational interventions on practice behaviors and patient outcomes. Additional limitations include a low survey response rate and a relatively short period of interaction and evaluation.

## CONCLUSION

In conclusion, multicomponent critical care tele-education in response to the COVID-19 global pandemic was feasible, inexpensive, and associated with a high level of clinician engagement and satisfaction. The intervention assessed in this study offers an excellent model for rapid, nimble, and large-scale distance learning programs for healthcare providers and can be especially valuable in low- and middle-income countries where critical care expertise is lacking.
